# Identifying SNAREs by Incorporating Deep Learning Architecture and Amino Acid Embedding Representation

**DOI:** 10.3389/fphys.2019.01501

**Published:** 2019-12-10

**Authors:** Nguyen Quoc Khanh Le, Tuan-Tu Huynh

**Affiliations:** ^1^Professional Master Program in Artificial Intelligence in Medicine, Taipei Medical University, Taipei, Taiwan; ^2^Department of Electrical Electronic and Mechanical Engineering, Lac Hong University, Bien Hoa, Vietnam; ^3^Department of Electrical Engineering, Yuan Ze University, Taoyuan, Taiwan

**Keywords:** SNARE proteins, deep learning, convolutional neural networks, word embedding, skip-gram

## Abstract

SNAREs (soluble N-ethylmaleimide-sensitive factor activating protein receptors) are a group of proteins that are crucial for membrane fusion and exocytosis of neurotransmitters from the cell. They play an important role in a broad range of cell processes, including cell growth, cytokinesis, and synaptic transmission, to promote cell membrane integration in eukaryotes. Many studies determined that SNARE proteins have been associated with a lot of human diseases, especially in cancer. Therefore, identifying their functions is a challenging problem for scientists to better understand the cancer disease as well as design the drug targets for treatment. We described each protein sequence based on the amino acid embeddings using fastText, which is a natural language processing model performing well in its field. Because each protein sequence is similar to a sentence with different words, applying language model into protein sequence is challenging and promising. After generating, the amino acid embedding features were fed into a deep learning algorithm for prediction. Our model which combines fastText model and deep convolutional neural networks could identify SNARE proteins with an independent test accuracy of 92.8%, sensitivity of 88.5%, specificity of 97%, and Matthews correlation coefficient (MCC) of 0.86. Our performance results were superior to the state-of-the-art predictor (SNARE-CNN). We suggest this study as a reliable method for biologists for SNARE identification and it serves a basis for applying fastText word embedding model into bioinformatics, especially in protein sequencing prediction.

## Introduction

**FIGURE 1:**
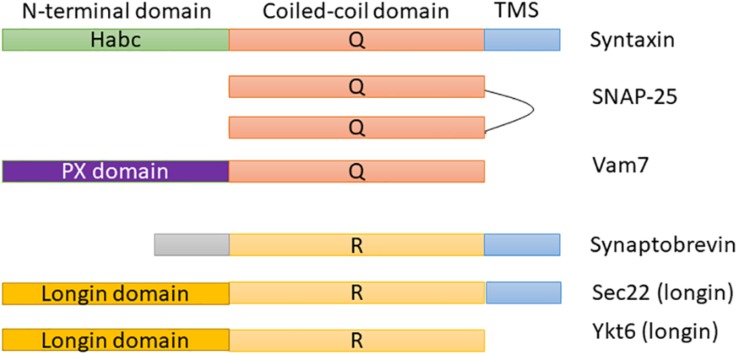
Domain architecture model of SNARE proteins.

Soluble N-ethylmaleimide-sensitive factor activating protein receptors (SNAREs) are the most important and broadly studied proteins in membrane fusion, trafficking, and docking. They are membrane-associated proteins that consist of distinguishing SNARE domains: heptad restates ∼60 amino acids in length that are predicted to assemble coiled-coils (Duman and Forte, 2003). Most SNAREs consist of only one SNARE motif adjacent to a single C-terminal membrane (e.g., syntaxin 1 and synaptobrevin 2). Figure 1 shows the domain architecture of some example SNAREs (e.g., syntaxin, SNAP-25, or Vam 7). As shown in these proteins, SNAREs generally consist of a central “SNARE domain” that is flanked by a variable N-terminal domain and a C-terminal single α-helical transmembrane anchor (Ungermann and Langosch, 2005). SNARE proteins are crucial for a broad range of cell processes, e.g., cytokinesis, synaptic transmission, and cell growth, to promote cell membrane integration in eukaryotes (Jahn and Scheller, 2006; Wickner and Schekman, 2008). There are two categories of SNARE: v-SNAREs incorporated into the membranes of transport vesicles during budding, and t-SNAREs associated with nerve terminal membranes. Researchers have recently identified a lot of SNARE proteins in human and they demonstrated that there is a crucial link between SNARE proteins and numerous diseases [e.g., neurodegenerative (Hou et al., 2017), mental illness (Dwork et al., 2002), and especially cancer (Meng and Wang, 2015; Sun et al., 2016)]. As a detail, a 1 bp deletion in SNAP-29 causes a novel neurocutaneous syndrome (Sprecher et al., 2005), mutation in the b-isoform of neuronal SNARE synaptosomal-associated protein of 25 kDa (SNAP-25) results in both diabetes and psychiatric disease (Jeans et al., 2007), mutations in VPS33B cause arthrogryposis–renal dysfunction–cholestasis (ARC) syndrome (Gissen et al., 2004), and so on.

Because SNARE proteins play an essential molecular function in cell biology, a wide variety of techniques were presented and used to investigate them. One of the best studies on SNAREs is molecular docking of synaptic vesicles with the presynaptic membrane in neurons. Another solution is to identify SNAREs from unknown sequence according to their motif information. In order to address it, Kloepper team is a first group that used bioinformatics techniques in this kind of problem. In their research, they have already built a database for retrieving and classifying SNARE proteins (Kloepper et al., 2007, 2008; Kienle et al., 2009). Furthermore, SNARE functions in sub-Golgi localization had also been predicted using bioinformatics techniques (van Dijk et al., 2008). Yoshizawa et al. (2006) identified SNAREs in membrane trafficking via extracting sequence motifs and the phylogenetic features. In the latest work, Le and Nguyen (2019) identified SNAREs by treating position-specific scoring matrices as images to feed into 2D convolutional neural network (CNN).

To our knowledge, only the study from Le and Nguyen (2019) conducted the SNARE protein prediction in membrane fusion by using machine learning techniques. However, their performance results need a lot of improvements, and we therefore motivate to create a better model for this. To address this, we transform the protein sequences into a continuous bag of nucleobases using fastText model (Bojanowski et al., 2017) and then carry out to identify them with the use of deep neural networks. Releasing by Facebook Research, fastText is a natural language processing (NLP) model for word embedding and text classification. It uses neural network for learning text representations and since its discovery, it has been used in a lot of different NLP problems (Joulin et al., 2017). It has been also used in interpreting biological sequences such as DNA sequences (Le, 2019; Le et al., 2019b) and protein sequences (Asgari et al., 2019), and here we provide a different application with a more in-depth analysis.

The idea is to treat protein sequence as a sentence and amino acids as words, we used fastText to train the language model on all sequences. Subsequently, this language model will be used to generate vectors for protein sequences. At the latest stage, we used a deep neural network to learn these vectors as features and perform supervised learning for classification. The rest of this paper is organized as follows: our materials and methods are introduced in the section “Methods”; some of our relevant experiments and results are introduced in the section “Results”; discussions of the model performance as well as limitations are given in the section “Discussion.”

## Methods

**FIGURE 2:**
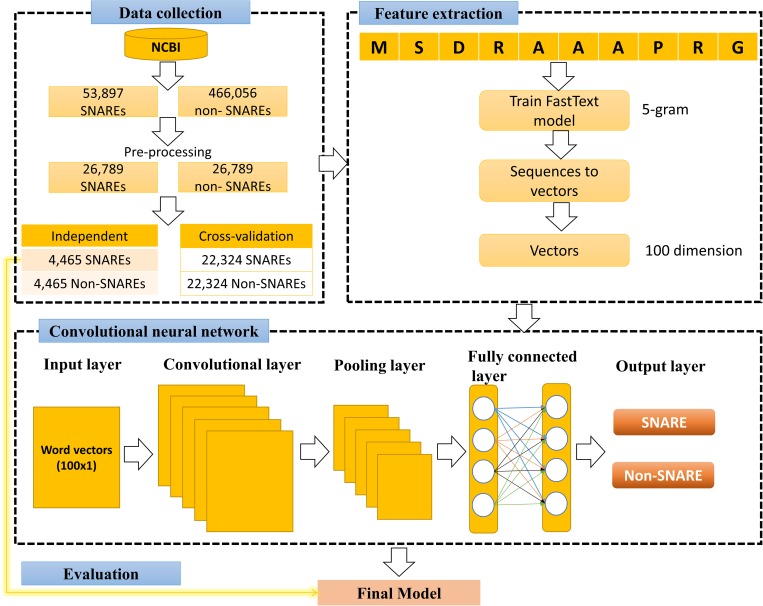
Flow chart of this study.

Figure 2 illustrates our flowchart which consists of three major processes: data collection, training fastText model and 1D CNN model. We describe the detailed description of our approach in the following paragraphs.

### Data Collection

The dataset retrieved from the National Center for Biotechnology Information (NCBI) (by 4-2-2019) (Coordinators, 2015), which is a large suite of online resources for biological information and data. Moreover, on-line resource conserved domain database (CDD) (Zheng et al., 2014) suggested that “SNARE superfamily” members could be identified using the SNARE motif “cl22856,” therefore, we used this information to generate non-redundant (annotated) SNARE proteins. This step ensures that we collected all corrected SNARE proteins including SNARE motif. There are many protein sources in NCBI, and we chose to collect all protein sequences from RefSeq (Pruitt et al., 2006). Next, to prevent overfitting problem, we used CD-HIT (Fu et al., 2012) to eliminate the redundant sequences with similarity greater than 30%, and the rest of proteins reaches 26,789 SNAREs. We used full sequences of proteins, thus it includes typical coiled coil as well as other motifs.

In the next step, we collected a negative set to treat our problem as a binary classification between positive (SNAREs) and negative set. To perform this, we retrieved all general proteins without the SNARE motif and with similarity more than 30%. Because the number of negative data was much higher than the number of positive data, it will cause difficulties in machine learning problem. Therefore, we randomly selected 26,789 negative samples to give balance training in our problem.

### Amino Acid Embedding Representation

Encouraged by the high performance of word embedding in many NLP tasks, we presented a similar feature set called “amino acid embedding.” The objective is to apply recent NLP models into biological sequences. It was first proposed by Asgari and Mofrad (2015) and successfully used to solve the latter biological problems related to sequence information (Habibi et al., 2017; Vang and Xie, 2017; Öztürk et al., 2018). Nevertheless, with the use of Word2Vector to describe the biological sequences, these findings had some disadvantages such as out-of-vocabulary cases for unknown words as well as not taking care of the inner structure of words. Accordingly, a critical issue therefore needs to be resolved is that instead of using an single specific vector representation for the protein word, the internal structure of each word needs to be taken into account. Facebook suggested fastText, which is a Word2vec extension that can handle the word as a continuous bag of character n-grams (Bojanowski et al., 2017), to perform this task. The vector for a word therefore consists of the number of n-grams of this type. It has been shown that fastText was more accurate than using Word2vec in a variety of fields (Joulin et al., 2017). Inspired by its accomplishments, previous researchers used it to describe biological sequences such as DNA enhancer sequence (Le et al., 2019b), DNA N6-methyladenine sites (Le, 2019) and protein sequence (Asgari et al., 2019).

The goal of this step is to encode nucleotides by establishing their vector space distribution, enabling them to be adopted by supervised learning algorithms. To perform a supervised learning classification, we need a set of features having the same dimension. Nonetheless, our protein sequences are of different lengths, so to address this issue, we set the embedding vector dimension to 100. This means that each protein sequence is represented as real numerical values of 100 and can be fed directly without pre-processing into any machine learning classifier. We have more special features for a good prediction by bringing this information into the dataset.

### Convolutional Neural Network

Convolutional neural network generally consists of multiple layers with each layer performing a particular function of translating its output into a functional representation. All layers are combined to form the architecture of our CNN system using a specific order. Similar to many published works in this field (Le et al., 2018, 2019a,c; Nguyen et al., 2019), different layers used in CNN for the current study include:

(1) Input layer of our CNN is a 1D vector, which is a vector of size 1 × 100 (created by fastText model).
(2) Convolutional layers were used as convolution operations to extract features embedded in the 1D input vector. These layers took a sliding window with specific stride shifting across all the input shapes. After sliding, the input shapes will be transformed into representative values. The spatial relationship between numeric values in the vectors has been preserved in this convolutional process. It will help this layer learn the important features using small slides of input data. Since the input of our CNN model is a vector of small size, we used the kernel size of 3 to deduce more information. This number of kernel has been used in previous works on CNN (Le et al., 2017, 2018, 2019a).
(3) Activation layer was performed after convolutional layers. It is an additional non-linear operation, called ReLU (Rectified Linear Unit) and is calculated as follows:
  (1) f⁢(x)=max⁡(0,x) 
  Where *x* is the number of inputs in a neural network. The purpose of ReLU is to introduce non-linearity in our CNN and help our model learn better from the data.
(4) Pooling layer was applied in convolutional layers to reduce the computational size for the next layers. There are three types of pooling layers, and we selected max pooling in our architecture to select the maximum value over a window of 2.
(5) Dropout layer was applied aiming to reduce the overfitting of our model and also to improve the performance results in some cases (Srivastava et al., 2014).
(6) Flatten layer was used to transform the input matrix into a vector. It always stand before fully connected layers.
(7) Fully connected layer was usually applied in the last stages of neural network architectures. In this layer, each node is fully connected with all the nodes of the previous layers. Two fully connected layers have been included in the current model. The first one connected all the input nodes to the flatten layer to help our model to gain more knowledge and perform better. This one was then connected to the output layer by the second layer. The number of nodes in the output layer is equal to 2 as identifying SNARE proteins was as a binary classification problem.
(8) Softmax was an evaluation function standing at the output of the model to determine the probability of each possible output. Its function could be calculated by the formula:
  (2) $$\sigma \,{\left(z\right)}_{i}=\frac{e^{z_{i}}}{\sum_{k=1}^{K}e^{z_{k}}}\;$$
  where *z* indicates the input vector with K-dimensional vector, σ(*z*)i is real values in the range (0, 1) and ith class is the predicted probability from sample vector *x*.

### Assessment of Predictive Ability

We firstly trained the model on the entire training set using 5-fold cross-validation technique. Since every 5-fold cross-validation produces different results each time, we performed ten times 5-fold cross-validation to achieve more reliable results. Thereafter, we reported the cross-validation performance by averaging all the ten times cross-validation tests. In the training process, hyper-parameter optimization has been used to identify the best parameters for each dataset. Finally, an independent test was applied to evaluate the performance and to ensure preventing any systematic bias in the cross-validation set.

Moreover, to evaluate the performance of our method, we applied Chou’s criterion (Chou, 2001) used in many bioinformatics studies. With this criterion, some standard metrics sensitivity, specificity, accuracy and Matthews correlation coefficient (MCC) are as follows:

(3) $$\mathrm{Sensitivity}=1-\frac{N_{-}^{+}}{N^{+}},0\leq \mathrm{Sen}\leq 1\;$$

(4) $$\mathrm{Specificity}=1-\frac{N_{+}^{-}}{N^{-}},0\leq \mathrm{Spec}\leq 1\;$$

(5) $$\mathrm{Accuracy}=1-\frac{N_{-}^{+}+N_{+}^{-}}{N^{+}+N^{-}},0\leq \mathrm{Acc}\leq 1\;$$

$$\mathrm{MCC}=\frac{1-\left(\frac{N_{-}^{+}}{N^{+}}+\frac{N_{+}^{-}}{N^{-}}\right)}{\sqrt{\left(1+\frac{N_{+}^{-}-N_{-}^{+}}{N^{+}}\right)\,\left(1+\frac{N_{-}^{+}-N_{+}^{-}}{N^{-}}\right)}},$$

(6) -1≤MCC≤1    

The relations between these symbols and the symbols in Eqs. (3–6) are given by:

$$\left\{ \begin{array}{c} N_{+}^{-}=\mathrm{FP}\\ N_{-}^{+}=\mathrm{FN}\\ \begin{array}{c} N^{+}=\mathrm{TP}+N_{-}^{+}\\ N^{-}=\mathrm{TN}+N_{+}^{-} \end{array} \end{array}\right.\;\left(7\right)$$

Where TP, FP, TN, FN are true positive, false positive, true negative, and false negative values, respectively.

## Results

### Composition of Amino Acid Representation in SNAREs and Non-SNAREs

**FIGURE 3:**
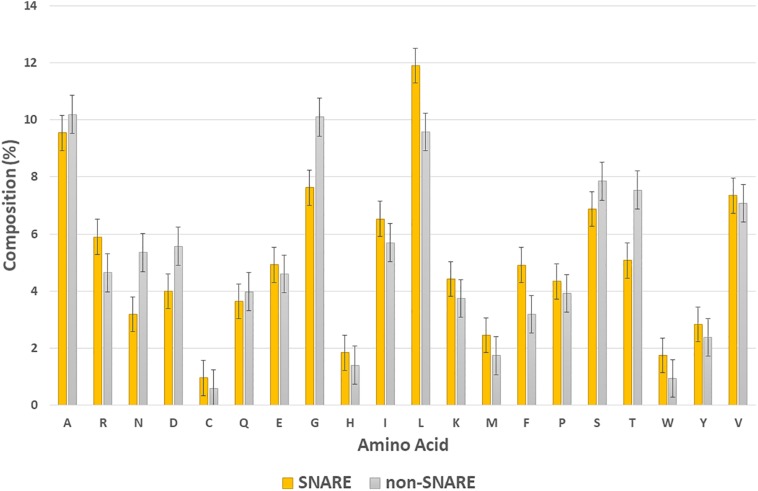
Composition of amino acid in SNAREs and non-SNAREs.

In this section, we would like to analyze the differences between SNARE and non-SNARE sequences in our dataset by computing the composition of amino acid representation between them. The amino acids which had the highest frequency in the positive and negative set are shown in Figure 3. It is easy to point out some of the differences between the two types of dataset. For instance, we were aware of the higher frequency of amino acid L, and F, and R in the SNARE proteins but lower in the non-SNAREs. Otherwise, the amino acids that appeared a lot in non-SNARE sequences are G, T, N, and D. Besides, we plotted the standard error bars at each column to statistically see the differences among amino acid compositions. These error bars aim to calculate confidence intervals, or margins of error to quantify uncertainty. As shown in Figure 3, there are some amino acids had significantly differences (with no overlap error bars) such as N, D, G, L, F, and T. Therefore, these amino acids might play a crucial role in identifying SNARE sequences and they can be special features that help our model predict SNAREs with high accuracy. This finding also plays an important role in further research that aims to analyze the motif information in SNARE proteins.

### Hyperparameters Optimization

Hyper-parameters are architecture-level parameters and are different from parameters of a model trained via backpropagation. To tune hyperparameters, we used the approach to choose a set of hyperparameters for speeding up the training process as well as preventing overfitting. As suggested by Chollet (2015), each step of the above hyper-parameter-tuning approach was integrated into the hyper-parameter-tuning process as follows:

• Selecting a specific set of hyper-parameters.
• Creating the model according to the specific set.
• Evaluating the performance results using testing dataset.
• Moving to the next set of hyper-parameters.
• Repeating.
• Measuring performance results on an independent dataset.

Keras framework library (Chollet, 2015) with a TensorFlow backend (Abadi et al., 2016) was used as a deep learning framework to build the 1D CNN architecture. We performed grid search on training set and used accuracy to select the next set of hyperparameters. Furthermore among the six optimizers in Keras [e.g., Adam, Adadelta, Adagrad, Stochastic Gradient Descent (SGD), RMSprop, and Adamax], Adadelta has given a superior performance. Therefore, we used Adadelta in our model to achieve an optimal result. This point is also proven in the previous protein function prediction using CNN (Le et al., 2017; Nguyen et al., 2019).

### SNARE Identification With Different n-Gram Levels

After tuning the optimal parameters for 1D CNN model, we evaluated the performance of this architecture on the datasets of different n-gram levels (from 1 to 5). In this step, all the measurement metrics were used to evaluate the comparative performance in both cross-validation and independent test. The result is displayed in Table 1. Table 1 shows that the performance results of n-gram levels are proportional. We were not able to achieve the best performance unless we used high levels of n-gram values. To maximize the performance of our models, we should choose the n-gram levels from 4 (accuracy of more than 97%). This means that the model only captures the special information in a high level of n-gram, increasing high level of n-gram will help to increase much in the results. In this study, we chose n-gram = 5 with the best metrics (accuracy of 97.5 and 92.8% in the cross-validation and independent test, respectively) to perform further experiments.

**TABLE 1:** Performance results on identifying SNAREs with different n-gram levels.

	**Cross validation**				**Independent**			
		
**n-gram**	**Sens**	**Spec**	**Acc**	**MCC**	**Sens**	**Spec**	**Acc**	**MCC**
1	83.8	88.7	86.3	0.73	39.4	94.6	67	0.41
2	93.7	91.6	92.6	0.85	83.1	87.4	85.2	0.71
3	95.8	97.6	96.7	0.93	87.4	95	91.2	0.83
4	96.7	98.1	97.4	0.95	88.7	96.4	92.6	0.85
5	96.6	98.4	97.5	0.95	88.5	97	92.8	0.86

In most of the supervised learning problems, our model can perform well during training test, but worse in another invisible data. This is called overfitting and our study, no exception also included in this issue. Therefore, an independent test was used in our study to ensure that our model also works well in a blind dataset with unseen data. As described in the previous part, our independent dataset contained 4,465 SNAREs and 4,465 non-SNAREs. None of these samples occur in the training set. As shown in Table 1, our independent testing results also comply with cross-validation results in most metrics. To detail, our independent testing performance achieved the accuracy of 92.8%, sensitivity of 88.5%, specificity of 97%, and MCC of 0.86. There is a very few overfitting in our model and it can demonstrate that our model has been well done in this type of dataset. Another reason is the use of dropout inside CNN structure and it helps us prevent overfitting.

### Comparative Performance Between Proposed Method and the Existing Methods

From the previous section, we chose the combination of 1D CNN and 5-gram as our optimal model for SNARE identification. In this section, we aim to compare the effectiveness of our proposed features with other research groups studying the same problem. As mentioned in the literature review, there have been some published works on identifying SNARE proteins using computational techniques. However, among of them, there is only one predictor to propose the machine learning techniques on predicting SNARE (Le and Nguyen, 2019). Therefore, we compared our performance with them in both cross-validation and independent test. Table 2 shows the performance results by highlighting the higher values for each metrics. It is clear that on average, our method outperforms the previous model in all measurement metrics. Therefore, we are able to generate effective features for identifying SNAREs with a better performance than PSSM profiles which had been used in the previous work.

**TABLE 2:** Comparative performance of predicting SNAREs between the proposed method and the previous published work.

	**Cross validation**				**Independent**			
		
**Predictor**	**Sens**	**Spec**	**Acc**	**MCC**	**Sens**	**Spec**	**Acc**	**MCC**
SNARE-CNN	76.6	93.5	89.7	0.7	65.8	90.3	87.9	0.46
Ours	**96.6**	**98.4**	**97.5**	**0.95**	**88.5**	**97**	**92.8**	**0.86**

*The bold values is to show the significant values for each metric.*

## Discussion

Based on the outstanding results of word embeddings in NLP, applying it to protein function prediction is an essential concern for biological researchers. In this study, we have approached a method using word embedding and deep learning for identifying SNARE proteins. Our structure is a combination between fastText (to train vectors model) and 1D CNN (to train deep learning model from the generated vectors). By using fastText, the protein sequences have been interpreted via different representations and we could generate the hidden information of them. While the other NLP models do not have sub-word information, it is an advantage of fastText that can help to improve this problem. Benefits of fastText when comparing to the other features have been also proven in the previous works based on their results (Do and Khanh Le, 2019; Le, 2019; Le et al., 2019b). We used 5-fold cross-validation set to train our model and an independent set to examine the performance results. Compared to the state-of-the-art predictor, our method produced superior performance in all the typical measurement metrics. Through this study, biologists can use our model to identify SNARE proteins with high accuracy and use them as necessary information for drug development. In addition, we contribute a method to interpret the information of protein sequences and further research is able to apply in bioinformatics research, especially in protein function prediction.

Furthermore, we provided our source codes and datasets at https://github.com/khanhlee/fastSNARE. The readers and biologists are able to reproduce our results as well as perform their classifications according our method. We also hope that our future research would be able to provide a web-server for the method of prediction as presented in this paper. Moreover, a limitation of using language model is that it could not consider mutations and SNPs in SNARE sequence. Therefore, further studies could integrate these information into fastText model to improve the predictive performance.

## Data Availability Statement

The raw data supporting the conclusions of this article will be made available by the authors, without undue reservation, to any qualified researcher.

## Author Contributions

Both authors conceived the ideas, designed the study, participated in the discussion of the results and writing of the manuscript, and read and approved the final version of the manuscript. NL conducted the experiments and analyzed the results.

## Conflict of Interest

The authors declare that the research was conducted in the absence of any commercial or financial relationships that could be construed as a potential conflict of interest.
